# Supplementary material for: A UMLS-based spell checker for natural language processing in vaccine safety
Source: BMC Med Inform Decis Mak. 2007 Feb 12;7:3. doi: 10.1186/1472-6947-7-3 (PMC1805499; doi:10.1186/1472-6947-7-3)
Supplement: Additional file 13 — RAP application directory. Sets up directory for RAP (RDF application for PHP) source code [file 1472-6947-7-3-S13.gz › rap/doc/phpdoc/resModel/ResList.html]

xml version="1.0" encoding="iso-8859-1"?


Docs For Class ResList


## Class ResList

Description

Description |
Methods (details)

Implementation of an rdf:Collection (rdf:List) Provides a convenience encapsulation for lists formed from chains of RDF statements arranged to form a head/tail cons-cell structure.

A well-formed list has cells that are made up of three statements: one denoting the rdf:type of the list cell, one denoting the link to the value of the list at that point, and one pointing to the list tail. If a list cell is not well-formed, list operations may fail in unpredictable ways. However, to explicitly check that the list is well-formed at all times is expensive, but you can call the isValid() method to manually check, if the list is well formed.

History:

- access: public
- todo: nothing
- author: Daniel Westphal <at d-westphal dot de mail at d-westphal dot de>
- version: V0.9.1

Located in /resModel/ResList.php (line 32)

```
Object
   |
   --Node
      |
      --Resource
         |
         --ResResource
            |
            --ResList
```

Method Summary

Description |
Methods (details)

ResList
ResList
([string $uri = null])

boolean
add
(object ResResource $resource)

object ResList
append
(mixed $resList, object ResList $ResList)

void
concatenate
(object ResList $ResList)

object ResList
cons
(object ResResource $value)

boolean
contains
(object ResResource $value)

object ResList
copy
()

ResResource
get
(integer $position)

array
getContentInArray
()

ResResource
getHead
()

object ResList
getTail
()

integer
indexOf
(object ResResource $resource, integer $offset)

integer
isEmpty
(object Statement $statement)

boolean
isValid
()

object ResList
remove
(object ResResource $value)

ResList
removeHead
()

boolean
removeList
()

object ResResource
replace
(integer $index, object ResResource $resource)

boolean
sameListAs
(object ResList $resList)

ResResource
setHead
(object ResResource $value)

object Reslist
setTail
(object ResList $resList)

Methods

Description |
Methods (details)

Constructor ResList (line 63)

Constructor You can supply a URI

- access: public

ResList

ResList
([string $uri = null])

- string
  $uri

add (line 103)

Add the given value to the end of the list.

it is only defined if this is not the empty list.

- access: public

boolean

add
(object ResResource $resource)

- object ResResource
  $resource

append (line 381)

Answer a new list that is formed by adding each element of

this list to the head of the given list. This is a non side-effecting operation on either this list or the given list, but generates a copy of this list. For a more storage efficient alternative, see concatenate

- access: public

object ResList

append
(mixed $resList, object ResList $ResList)

- object ResList
  $ResList

concatenate (line 360)

Change the tail of this list to point to the given list, so that this list becomes the list of the concatenation of the elements of both lists. This is a side-effecting operation on this list; for a non side-effecting alternative, see append.

- access: public

void

concatenate
(object ResList $ResList)

- object ResList
  $ResList

cons (line 424)

Return a reference to a new list cell whose head is value and whose tail is this list.

- access: public

object ResList

cons
(object ResResource $value)

- object ResResource
  $value

contains (line 253)

Answer true if the given node appears as the value of a value of any of the cells of this list.

- access: public

boolean

contains
(object ResResource $value)

- object ResResource
  $value

copy (line 403)

Answer a list that contains all of the elements of this list in the same order, but is a duplicate copy in the underlying model.

- access: public

object ResList

copy
()

get (line 80)

Returns the value of the list element at the specified position or null.

- access: public

ResResource

get
(integer $position)

- integer
  $position

getContentInArray (line 324)

Get all values in the list as an array of ResResources

- access: public

array

getContentInArray
()

getHead (line 162)

Get the value that is associated with the head of the list.

- access: public

ResResource

getHead
()

getTail (line 266)

Get the list that is the tail of this list.

- access: public

object ResList

getTail
()

indexOf (line 201)

Get the Position of the first occurrence of the given value in the list, or -1 if the value is not in the list.

You can supply an offset to search for values. (First element has offset 0) Default is 0

- access: public

integer

indexOf
(object ResResource $resource, integer $offset)

- object ResResource
  $resource
- integer
  $offset

isEmpty (line 313)

Returns true, if this list is empty

- access: public

integer

isEmpty
(object Statement $statement)

- object Statement
  $statement

isValid (line 545)

Answer true if the list is well-formed, by checking that each node is correctly typed, and has a head and tail pointer from the correct vocabulary

- access: public

boolean

isValid
()

remove (line 506)

Remove the given value from this list.

If value does not occur in the list, no action is taken. Since removing the head of the list will invalidate the list head cell, in general the list must return the list that results from this operation. However, in many cases the return value will be the same as the object that this method is invoked on.

- access: public

object ResList

remove
(object ResResource $value)

- object ResResource
  $value

removeHead (line 175)

Remove the head of the list. The tail of the list remains in the model. Note that no changes are made to list cells that point to this list cell as their tail.

- access: public

ResList

removeHead
()

removeList (line 284)

Remove all of the components of this list from the model.

Note that this is operation is only removing the list cells themselves, not the resources referenced by the list - unless being the object of an rdf:first statement is the only mention of that resource in the model.

- access: public

boolean

removeList
()

replace (line 231)

Replace the value at the i'th position in the list with the given value

- access: public

object ResResource

replace
(integer $index, object ResResource $resource)

- integer
  $index
- object ResResource
  $resource

sameListAs (line 471)

Answer true if this list has the same elements in the same order as the given list. Note that the standard equals test just tests for equality of two given list cells.

While such a test is sufficient for many purposes, this test provides a broader equality definition, but is correspondingly more expensive to test.

- access: public

boolean

sameListAs
(object ResList $resList)

- object ResList
  $resList

setHead (line 142)

Update the head of the list to have the given value, and return the previous value.

- access: public

ResResource

setHead
(object ResResource $value)

- object ResResource
  $value

setTail (line 446)

Update the list cell at the front of the list to have the given list as tail. The old tail is returned, and remains in the model.

- access: public

object Reslist

setTail
(object ResList $resList)

- object ResList
  $resList

#### Inherited Methods


Inherited From ResResource

> ResResource::ResResource()  
> ResResource::addProperty()  
> ResResource::equals()  
> ResResource::getAssociatedModel()  
> ResResource::getIsAnon()  
> ResResource::getProperty()  
> ResResource::hasProperty()  
> ResResource::listProperties()  
> ResResource::removeAll()  
> ResResource::removeProperties()  
> ResResource::setAssociatedModel()  
> ResResource::setIsAnon()  
> ResResource::setURI()

Inherited From Resource

> Resource::Resource()  
> Resource::equals()  
> Resource::getLabel()  
> Resource::getLocalName()  
> Resource::getNamespace()  
> Resource::getURI()  
> Resource::toString()

Inherited From Object

> Object::toString()

Documentation generated on Thu, 7 Jul 2005 13:43:37 +0200 by phpDocumentor 1.3.0RC3
